# P-1332. Estimates of Waning Influenza Vaccine Effectiveness Against Laboratory-Confirmed Influenza in California, October 2023–March 2024

**DOI:** 10.1093/ofid/ofae631.1510

**Published:** 2025-01-29

**Authors:** Sophie Zhu, Joshua Quint, Tomás M León, Monica Sun, Nancy Li, Mark W Tenforde, Seema Jain, Robert Schechter, Cora Hoover, Erin Murray

**Affiliations:** Centers for Disease Control and Prevention, Richmond, California; California Department of Public Health, Richmond, California; California Department of Public Health, Richmond, California; California Department of Public Health, Richmond, California; California Department of Public Health, Richmond, California; US Centers for Disease Control and Prevention, Decatur, Georgia; California Department of Public Health, Richmond, California; California Department of Public Health, Richmond, California; California Department of Public Health, Richmond, California; California Department of Public Health, Richmond, California

## Abstract

**Background:**

Annually, an estimated 9.3 to 41 million persons experience influenza illness in the United States. Vaccination protects against influenza and associated complications, and vaccine effectiveness (VE) varies between and within seasons. Identifying declines in VE can guide appropriate public health actions. Mandatory influenza vaccine reporting to California’s immunization information system began January 1, 2023, and all influenza laboratory testing results, including negative results, were reportable starting June 15, 2023. We calculated VE of seasonal influenza vaccines against laboratory-confirmed influenza over time using public health data reported to the California Department of Public Health (CDPH).

Influenza vaccine effectiveness by time since vaccination (days) using mandatory reported California influenza laboratory and immunization data, October 2023–March 2024

**Figure:**
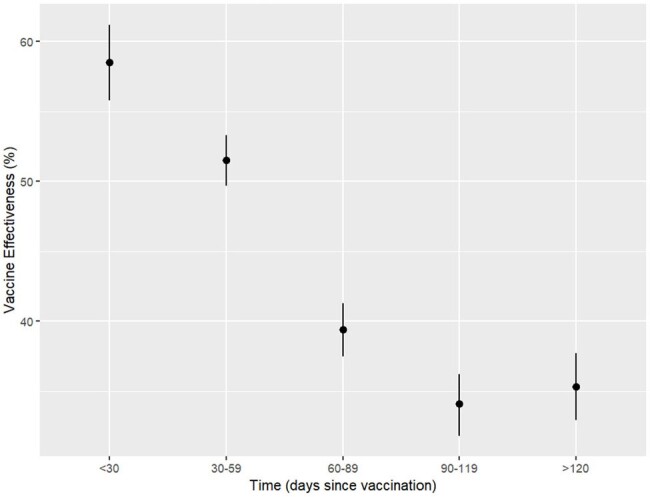

**Methods:**

We included influenza laboratory surveillance data for all persons aged ≥6 months during October 1, 2023–March 31, 2024, and immunization data during August 1, 2023–March 31, 2024. VE for laboratory-confirmed influenza was calculated using a case control design as 100% x (1 – adjusted odds ratio [aOR]); aOR was odds ratio of vaccination among case-patients that received a positive influenza test result versus control-patients that received a negative influenza test result. Using mixed-effects logistic regression, estimates were adjusted for age, ethnicity, and race as fixed effects; specimen week and county of residence were random effects. Estimates were stratified by time since vaccination.

**Results:**

Overall, 18% of case-patients were vaccinated versus 31% of control-patients. Median time since vaccination was 85 days (range: 14–241 days) for cases and 87 days (range: 14–241 days) for controls. VE through March 31, 2024, was 43% against laboratory-confirmed influenza among persons aged ≥6 months. VE was 59% for persons vaccinated 14–29 days before their influenza test result; 52% for 30–59 days; 39% for 60–89 days; 34% for 90–119 days; and 35% for ≥120 days. Stratified by calendar month of testing, VE similarly declined with increasing time since vaccination.

**Conclusion:**

Influenza vaccination reduced the risk of testing positive for influenza, but VE declined with time after vaccination. Timing immunization for maximum protection during periods of higher risk, along with additional preventive measures, might further reduce incidence.

**Disclosures:**

**All Authors**: No reported disclosures

